# Supplementary material for: DNN-m6A: A Cross-Species Method for Identifying RNA N6-methyladenosine Sites Based on Deep Neural Network with Multi-Information Fusion
Source: Genes (Basel). 2021 Feb 28;12(3):354. doi: 10.3390/genes12030354 (PMC7997228; doi:10.3390/genes12030354)
Supplement: Supplementary file 1 [file genes-12-00354-s001.pdf]

Supplementary Materials:

DNN-m6A: A Cross-Species Method for Identifying RNA N6-methyladenosine Sites Based on Deep Neural Network with Multi-Information Fusion

Lu Zhang[1], Xinyi Qin[1], Min Liu[1], Ziwei Xu[2], and Guangzhong Liu[1,*]

[1] College of Information Engineering, Shanghai Maritime University, Shanghai 201306, China
[2] Polytech Nantes, Bâtiment Ireste, Nantes 44300, France
* Correspondence: gzhliu@shmtu.edu.cn

Table of Contents

Supplementary Tables

Table S1. ACC values corresponding to different λ and w in PseDNC.

Table S2. ACC values corresponding to different K_{max} in KSNPEs.

Table S3. ACC values corresponding to different feature extraction methods (All represents the initial feature space corresponding to the fusion of eight features).

Table S4. Performance of different feature selection methods.

Table S5. Performance comparison with iRNA-m6A on the training datasets.

Table S6. Performance comparison with iRNA-m6A on the independent datasets.

Table S7. Comparison of prediction results of different methods on S51.

1. Supplementary Tables

Table S1.

ACC values corresponding to different λ and w in PseDNC.

λ	w	H_B	H_K	H_L	M_B	M_H	M_K	M_L	M_T	R_B	R_K	R_L
λ=10	w =0.1	0.6691	**0.7671**	0.7614	0.7662	0.6954	0.7860	0.6752	0.7173	0.7277	0.8031	0.7934
	w =0.3	0.6662	0.7654	0.7610	0.7660	0.6981	0.7838	0.6754	0.7163	0.7238	0.8018	0.7926
	w =0.5	0.6673	0.7658	0.7618	**0.7677**	0.6963	0.7822	0.6755	0.7160	0.7236	0.8013	0.7929
	w =0.7	0.6661	0.7656	0.7606	0.7665	0.6979	0.7831	0.6748	0.7173	0.7226	0.8013	0.7929
	w =0.9	0.6654	0.7653	0.7621	0.7672	0.6972	0.7835	0.6742	0.7156	0.7236	0.8018	0.7920
λ=20	w =0.1	0.6734	0.7645	0.7587	0.7647	0.6995	0.7861	0.6792	0.7171	0.7311	0.8045	0.7926
	w =0.3	0.6723	0.7662	0.7593	0.7647	0.6999	0.7854	0.6795	0.7179	0.7294	0.8040	0.7937
	w =0.5	0.6738	0.7657	0.7565	0.7640	0.6988	0.7859	0.6799	0.7178	0.7285	0.8048	0.7937
	w =0.7	0.6740	0.7645	0.7568	0.7655	0.6979	0.7867	0.6800	0.7175	0.7296	0.8050	**0.7943**
	w =0.9	0.6719	0.7644	0.7565	0.7646	0.6976	0.7861	**0.6803**	**0.7187**	0.7292	0.8059	0.7926
λ=30	w =0.1	**0.6756**	0.7663	0.7686	0.7650	0.7097	0.7869	0.6797	0.7177	0.7362	0.8067	0.7917
	w =0.3	0.6737	0.7651	**0.7713**	0.7654	0.7094	0.7884	0.6788	0.7179	0.7366	**0.8079**	0.7931
	w =0.5	0.6737	0.7655	0.7694	0.7642	0.7088	**0.7899**	0.6800	0.7181	**0.7377**	0.8066	0.7926
	w =0.7	0.6744	0.7633	0.7703	0.7650	**0.7097**	0.7891	0.6770	0.7179	0.7351	0.8059	0.7926
	w =0.9	0.6738	0.7640	0.7701	0.7642	0.7092	0.7890	0.6771	0.7181	0.7345	0.8060	0.7906

Table S2.

ACC values corresponding to different K_{max} in KSNPEs.

Parameter	H_B	H_K	H_L	M_B	M_H	M_K	M_L	M_T	R_B	R_K	R_L
K_{max}=1	0.6856	0.7691	0.7640	0.7667	0.7079	0.7926	0.6909	0.7260	0.7321	0.8098	0.7897
K_{max}=2	0.6940	0.7727	0.7732	0.7705	0.7142	0.7951	0.6972	**0.7389**	0.7445	0.8133	0.7965
K_{max}=3	**0.7014**	**0.7754**	0.7724	0.7713	0.7163	0.7970	**0.7023**	0.7368	0.7436	**0.8156**	**0.8076**
K_{max}=4	0.6995	0.7737	0.7762	0.7703	**0.7263**	0.7964	0.7006	0.7362	0.7426	0.8131	0.7997
K_{max}=5	0.6958	0.7750	**0.7794**	**0.7714**	0.7210	**0.7986**	0.7006	0.7333	**0.7474**	0.8107	0.8028

Table S3.

ACC values corresponding to different feature extraction methods (All represents the initial feature space corresponding to the fusion of eight features).

Methods	H_B	H_K	H_L	M_B	M_H	M_K	M_L	M_T	R_B	R_K	R_L
BE	0.6979	0.7728	0.7756	0.7626	0.7001	0.7864	0.6793	0.7217	0.7279	0.7986	0.7775
KSNPFs	0.7014	0.7754	0.7794	0.7714	**0.7263**	**0.7986**	0.7023	0.7389	0.7474	0.8156	**0.8076**
ENAC	0.7027	0.7789	0.7728	0.7665	0.7065	0.7909	0.6833	0.7207	0.7362	0.8066	0.7994
NCP	0.6974	0.7766	0.7840	0.7662	0.7006	0.7914	0.6857	0.7217	0.7336	0.8034	0.7875
PseDNC	0.6756	0.7671	0.7713	0.7677	0.7097	0.7899	0.6803	0.7187	0.7377	0.8079	0.7943
TNC	0.6996	0.7759	0.7864	0.7743	0.7204	0.7972	**0.7099**	**0.7394**	0.7457	**0.8159**	0.8053
PSNP	0.6947	0.7740	0.7872	0.7683	0.7040	0.7881	0.6795	0.7145	0.7370	0.8016	0.7948
PSDP	0.7064	0.7773	**0.7893**	0.7734	0.7233	0.7905	0.6986	0.7273	**0.7538**	0.8101	0.8042
All	**0.7115**	**0.7828**	0.7743	**0.7755**	0.7129	0.7905	0.7008	0.7387	0.7483	0.8053	0.7860

Table S4.

Performance of different feature selection methods.

Species	Tissues	Initial	Methods	Optimal	ACC	Sn	Sp	MCC	AUC
Human	Brain	690	LLE	82	0.6704	0.7585	0.5822	0.3475	0.7396
			mRMR		0.7152	0.8211	0.6093	0.4409	0.7899
			SE		0.7117	0.8089	0.6145	0.4322	0.7912
			SVD		0.7127	0.8085	0.6169	0.4338	0.7907
			EN		**0.7344**	0.8165	0.6523	0.4764	0.8131
	Kidney	670	LLE	91	0.7570	0.8201	0.6939	0.5187	0.8374
			mRMR		0.7965	0.8664	0.7265	0.5991	0.8764
			SE		0.7802	0.8415	0.7188	0.5668	0.8666
			SVD		0.7819	0.8380	0.7258	0.5687	0.8684
			EN		**0.7984**	0.8640	0.7328	0.6023	0.8826
	Liver	722	LLE	103	0.7709	0.7939	0.7479	0.5441	0.8435
			mRMR		0.8022	0.8466	0.7578	0.6078	0.8816
			SE		0.7918	0.8269	0.7566	0.5860	0.8692
			SVD		0.7887	0.8155	0.7619	0.5795	0.8688
			EN		**0.8077**	0.8466	0.7688	0.6188	0.8859
Mouse	Brain	702	LLE	114	0.7460	0.7598	0.7323	0.4943	0.8240
			mRMR		0.7845	0.8116	0.7574	0.5708	0.8690
			SE		0.7750	0.7858	0.7641	0.5521	0.8590
			SVD		0.7716	0.7855	0.7576	0.5446	0.8587
			EN		**0.7890**	0.8160	0.7621	0.5807	0.8758
	Heart	706	LLE	117	0.6956	0.7619	0.6293	0.3956	0.7592
			mRMR		0.7308	0.7824	0.6792	0.4657	0.8129
			SE		0.7133	0.7397	0.6870	0.4283	0.7989
			SVD		0.7215	0.7624	0.6806	0.4452	0.8035
			EN		**0.7565**	0.7865	0.7265	0.5144	0.8375
	Kidney	722	LLE	88	0.7785	0.8083	0.7488	0.5591	0.8561
			mRMR		0.8036	0.8513	0.7559	0.6110	0.8844
			SE		0.7964	0.8457	0.7470	0.5967	0.8790

			SVD		0.7985	0.8462	0.7508	0.6007	0.8799
			EN		**0.8151**	0.8530	0.7771	0.6331	0.8944
	Liver	680	LLE	86	0.6579	0.8004	0.5154	0.3312	0.7225
			mRMR		0.7054	0.8309	0.5800	0.4261	0.7820
			SE		0.6942	0.8270	0.5613	0.4034	0.7746
			SVD		0.6950	0.8123	0.5778	0.4023	0.7764
			EN		**0.7303**	0.8210	0.6397	0.4697	0.8114
	Testis	664	LLE	151	0.6974	0.7130	0.6818	0.3971	0.7689
			mRMR		0.7459	0.7604	0.7315	0.4932	0.8315
			SE		0.7368	0.7559	0.7177	0.4740	0.8168
			SVD		0.7455	0.7718	0.7191	0.4927	0.8244
			EN		**0.7616**	0.8007	0.7225	0.5259	0.8429
Rat	Brain	722	LLE	88	0.7158	0.7666	0.6650	0.4351	0.7981
			mRMR		0.7689	0.8099	0.7279	0.5400	0.8516
			SE		0.7479	0.7874	0.7083	0.4980	0.8362
			SVD		0.7579	0.7963	0.7194	0.5180	0.8412
			EN		**0.7819**	0.8180	0.7457	0.5657	0.8672
	Kidney	690	LLE	88	0.7859	0.8340	0.7378	0.5749	0.8649
			mRMR		0.8236	0.8430	0.8042	0.6485	0.9012
			SE		0.8121	0.8439	0.7804	0.6258	0.8937
			SVD		0.8143	0.8331	0.7955	0.6298	0.8945
			EN		**0.8321**	0.8488	0.8153	0.6658	0.9087
	Liver	680	LLE	87	0.7835	0.8411	0.7259	0.5713	0.8602
			mRMR		0.8156	0.8400	0.7911	0.6330	0.8902
			SE		0.8062	0.8400	0.7724	0.6143	0.8787
			SVD		0.7965	0.8269	0.7662	0.5949	0.8782
			EN		**0.8229**	0.8428	0.8031	0.6474	0.8962

Note: "Initial" represents the dimension of initial feature vector sets without doing feature reduction, "Optimal" denotes the dimension of the optimal feature subsets.

Table S5.

Performance comparison with iRNA-m6A on the training datasets.

Species	Tissues	Methods	ACC	Sn	Sp	MCC	AUC
Human	Brain	DNN-m6A	**0.7378**	0.7848	0.6908	0.48	0.8165
		iRNA-m6A	0.7126	0.7479	0.6619	0.41	0.7756
	Kidney	DNN-m6A	**0.8048**	0.8356	0.7739	0.61	0.8841
		iRNA-m6A	0.7899	0.8085	0.7634	0.57	0.8634
	Liver	DNN-m6A	**0.8130**	0.8219	0.8041	0.63	0.8905
		iRNA-m6A	0.8013	0.8132	0.7813	0.59	0.8738
Mouse	Brain	DNN-m6A	**0.7936**	0.8176	0.7697	0.59	0.8778
		iRNA-m6A	0.7875	0.7932	0.7690	0.58	0.8701
	Heart	DNN-m6A	**0.7617**	0.7751	0.7483	0.52	0.8439
		iRNA-m6A	0.7276	0.7524	0.6897	0.44	0.7948
	Kidney	DNN-m6A	**0.8196**	0.8320	0.8072	0.64	0.8953
		iRNA-m6A	0.7998	0.8260	0.7731	0.60	0.8726

	Liver	DNN-m6A	**0.7358**	0.7757	0.6959	0.47	0.8139
		iRNA-m6A	0.7059	0.7493	0.6559	0.41	0.7743
	Testis	DNN-m6A	**0.7662**	0.8099	0.7225	0.53	0.8493
		iRNA-m6A	0.7440	0.7814	0.7002	0.48	0.8156
Rat	Brain	DNN-m6A	**0.7827**	0.7908	0.7746	0.57	0.8678
		iRNA-m6A	0.7596	0.7700	0.7347	0.50	0.8282
	Kidney	DNN-m6A	**0.8338**	0.8427	0.8249	0.67	0.9104
		iRNA-m6A	0.8178	0.8246	0.8005	0.63	0.8877
	Liver	DNN-m6A	**0.8263**	0.8417	0.8110	0.65	0.8991
		iRNA-m6A	0.8090	0.8309	0.7633	0.60	0.8766

Table S6.

Performance comparison with iRNA-m6A on the independent datasets.

Species	Tissues	Methods	ACC	Sn	Sp	MCC	AUC
Human	Brain	DNN-m6A	**0.7327**	0.7502	0.7152	0.47	0.8147
		iRNA-m6A	0.711	0.6950	0.7298	0.42	0.7845
	Kidney	DNN-m6A	**0.7989**	0.8316	0.7662	0.60	0.8780
		iRNA-m6A	0.7776	0.7713	0.7842	0.56	0.8565
	Liver	DNN-m6A	**0.8096**	0.8178	0.8014	0.62	0.8854
		iRNA-m6A	0.7901	0.7819	0.7987	0.58	0.8681
Mouse	Brain	DNN-m6A	**0.7859**	0.7507	0.8211	0.57	0.8760
		iRNA-m6A	0.7826	0.7720	0.7941	0.57	0.8613
	Heart	DNN-m6A	**0.7511**	0.7727	0.7295	0.50	0.8338
		iRNA-m6A	0.713	0.7052	0.7213	0.43	0.7878
	Kidney	DNN-m6A	**0.8087**	0.8117	0.8057	0.62	0.8888
		iRNA-m6A	0.7931	0.7837	0.8032	0.59	0.8697
	Liver	DNN-m6A	**0.7295**	0.7639	0.6951	0.46	0.8079
		iRNA-m6A	0.6879	0.6782	0.6986	0.38	0.762
	Testis	DNN-m6A	**0.7712**	0.8009	0.7416	0.54	0.8535
		iRNA-m6A	0.7354	0.7219	0.7508	0.47	0.8182
Rat	Brain	DNN-m6A	**0.7799**	0.7767	0.7831	0.56	0.8624
		iRNA-m6A	0.7514	0.7393	0.7648	0.50	0.8265
	Kidney	DNN-m6A	**0.8304**	0.8534	0.8074	0.66	0.9109
		iRNA-m6A	0.8142	0.8018	0.8277	0.63	0.8968
	Liver	DNN-m6A	**0.8164**	0.8280	0.8048	0.63	0.8956
		iRNA-m6A	0.7985	0.7774	0.8231	0.60	0.8761

Table S7.

Comparison of prediction results of different methods on S51.

Methods	ACC	Sn	Sp	MCC
pRNAm-PC	69.74%	69.72%	69.75%	0.4000
M6AMRFS	74.25%	75.21%	73.30%	0.4852
iN6-Methyl (5-step)	75.38%	76.15%	74.62%	0.5078
**DNN-m6A**	**78.50%**	**78.66%**	**78.34%**	**0.5707**
